# A Semi-Automated Imaging Flow Cytometry Workflow for High-Throughput Quantification of Compound Internalization with IDEAS and FluoSta Software

**DOI:** 10.3390/mps8060138

**Published:** 2025-11-09

**Authors:** Kirill Elfimov, Ludmila Gotfrid, Alina Nokhova, Mariya Gashnikova, Dmitriy Baboshko, Aleksei Totmenin, Aleksandr Agaphonov, Natalya Gashnikova

**Affiliations:** 1 State Research Center of Virology and Biotechnology ‘Vector’, Koltsovo 630559, Russia; grushakova@inbox.ru (L.G.);; 2 Federal Research Center for Fundamental and Translational Medicine, Novosibirsk 630060, Russia

**Keywords:** imaging flow cytometry, internalization, statistical pipeline, cellular uptake, FluoSta

## Abstract

For many therapeutic agents to be effective against intracellular targets, they must first be able to penetrate the cell membrane. Current methodologies for assessing internalization, such as confocal microscopy and conventional flow cytometry, are limited by low throughput or an inability to provide precise spatial information on signal localization. Here, we present a comprehensive, semi-automated analytical pipeline for investigating compound internalization based on imaging flow cytometry, which is designed to address these limitations. Our workflow details the procedure from sample preparation and data acquisition on an Amnis FlowSight cytometer to analysis using IDEAS 6.2 software with a custom-designed template. Key features of our approach include the automated discrimination of signal between the plasma membrane and cytoplasmic compartments, the calculation of an internalization coefficient, and the introduction of a novel parameter—signal distribution entropy—to quantify the uniformity of the compound distribution within cells. For the statistical analysis, we developed FluoSta v1.0, a software tool that automates descriptive statistics and analysis of variance (ANOVA with Tukey’s post hoc test) and facilitates data visualization. The pipeline’s utility was demonstrated in a series of model experiments, including a comparative assessment of the internalization efficiency of PS- versus PS/LNA-modified compounds in MT-4 cell cultures.

## 1. Introduction

The efficacy of many contemporary therapeutic agents targeting intracellular targets (e.g., antisense oligonucleotides, siRNA, peptides, nanoparticles) is directly dependent on their ability to penetrate the cell membrane and reach the target internal compartment. For example, antisense oligonucleotides (ASOs) demonstrate high binding specificity to target nucleic acids [1,2]. However, they can degrade in endosomes under the action of enzymes without special chemical modifications (phosphorothioate, locked nucleic acid, etc.) [3], which can affect their bioavailability [4]. Similarly, many peptides and nanoparticles must traverse the plasma membrane barrier to exert their intended effects.

Conventional methods for evaluating internalization have their drawbacks. Confocal microscopy provides a high resolution and allows for the visualization of specific cellular compartments; however, it analyzes a limited number of cells (low statistical power) and is time-consuming. Conventional flow cytometry allows for the analysis of hundreds of thousands of cells but only records a fluorescence signal without information on its localization. To circumvent this limitation, the enzymatic treatment of cells is used to remove molecules adsorbed on the cell surface [5]. Currently, no standardized procedure exists for evaluating internalization, and each method has its own strengths and shortcomings.

Imaging flow cytometry effectively addresses the limitations of the aforementioned techniques by integrating high-throughput capabilities, thereby ensuring robust statistical power, with detailed visualization of every individual cell. This allows for the simultaneous analysis of both quantitative flow parameters and morphology of intracellular probe distribution. The application of image masks facilitates the automated discrimination of signal localization to specific subcellular compartments, such as the plasma membrane, cytoplasm, or nucleus. Furthermore, integrated software features enable quantitative co-localization analysis, which is essential for verifying the delivery of compounds to their intended intracellular targets. This methodology has been successfully employed in related contexts; for instance, Ofir-Birin et al. utilized imaging flow cytometry to monitor the active uptake of extracellular vesicles generated during infection with Plasmodium falciparum [6]. Similarly, Smirnov et al. applied a comparable approach to investigate the binding and internalization of Neisseria gonorrhoeae into human neutrophils [7].

Herein, we present a comprehensive pipeline for the acquisition, processing, and statistical analysis of internalization data for fluorescently labeled chemical compounds using the Amnis imaging flow cytometry platform (CytekBiosciences, Fremont, CA, USA). We demonstrate its application in an experiment investigating the uptake of PS- and PS/LNA-modified antisense oligonucleotides into cells of the human immortalized lymphoid line MT-4, as previously described in a preprint by Gotfrid et al. [8].

## 2. Materials and Methods

### 2.1. Cell Culture

The human immortalized lymphoid cell line MT-4 was used in this study. The cell line was obtained from the collection of the State Research Center of Virology and Biotechnology “Vector” (Rospotrebnadzor, Koltsovo, Russia). Cells were maintained in RPMI-1640 medium (Gibco, Waltham, MA, USA) supplemented with 10% heat-inactivated fetal bovine serum (Gibco, Waltham, MA, USA), 2 mM L-glutamine, and 20 μg/mL gentamicin. Cultures were grown in T-25 suspension flasks (Eppendorf, Hamburg, Germany) at 37 °C in a humidified atmosphere of 5% CO_2_. Cells were seeded at a density of 350,000 cells per mL of complete growth medium and subcultured every 3–4 days.

### 2.2. Antisense Oligonucleotides and Cell Treatment

Fully phosphorothioate-modified antisense oligonucleotides were studied, with select compounds containing LNA modifications at their 3′ and/or 5′ termini. All oligonucleotides were 5′-end labeled with a FAM fluorophore (Table 1).

Cells were seeded into culture flasks at 7 mL of growth medium per flask, with a concentration of 1 × 10^6^ cells/mL. The corresponding antisense oligonucleotides were added to each sample to produce a final concentration of 2 × 10^−4^ M. The incubation times were 1, 4, 12, 24, 36, and 48 h.

### 2.3. Imaging Flow Cytometry

The internalization capacity of the oligonucleotides was assessed using imaging flow cytometry on an Amnis FlowSight platform (CytekBiosciences, Fremont, CA, USA). Excitation of the FAM fluorophore conjugated to the oligonucleotides, the vital dye propidium iodide (PI), and brightfield imaging were performed using a 488 nm laser operating at 60 mW (with filter configurations of 532/55, 610/30, and 457/45, respectively). Images were acquired at a 20× overall magnification (objective numerical aperture = 0.6) and a pixel size of 1 × 1 μm.

### 2.4. Confocal Microscopy

Imaging was performed using an Olympus FV3000 laser scanning confocal microscope (Olympus, Tokyo, Japan). For image acquisition, 488 nm (ASO-FAM, 10% power) and 642 nm (CD4-AF700, BF, 50% power) lasers were employed. Detection was carried out using high-sensitivity detectors (HSD1 and HSD2) set at 500 V.

### 2.5. Required Reagents

- RPMI-1640 culture medium (Gibco, Waltham, MA, USA; Cat. No. 11875093).
- One Shot FBS (Gibco, Waltham, MA, USA; Cat. No. A5209502).
- Penicillin–Streptomycin (10,000 U/mL)—optional (Gibco, Waltham, MA, USA; Cat. No. 15140122).
- 1× DPBS, without Ca^2+^ and Mg^2+^ (Gibco, Waltham, MA, USA; Cat. No. 14190144). DPBS is preferred for working with live cells, although standard 1× PBS (Gibco, Waltham, MA, USA; Cat. No. 10010023) can be used as an alternative.
- Propidium iodide (PI), 0.1 µg/mL working solution (Lumiprobe, Hunt Valley, MD, USA; Cat. No. 19010).
- Microcentrifuge tubes (1.5 mL; Eppendorf, Hamburg, Germany; Cat. No. 022364111).
- Bovine Serum Albumin (BSA) (Thermo Fisher Scientific, Waltham, MA, USA; Cat. No. B14).
- Antibodies against CD4 or other surface markers (optional)—CD4 Monoclonal Antibody (RPA-T4), Alexa Fluor™ 700 (Thermo Fisher Scientific, Waltham, MA, USA; Cat. No. 56-0049-42).
- Antibodies against Clathrin Heavy Chain (optional)—Clathrin Heavy Chain Monoclonal Antibody (X22), Alexa Fluor™ 647 (Thermo Fisher Scientific, Waltham, MA, USA; Cat. No. MA1-065-A647).
- Fixation Buffer (BioLegend, San Diego, CA, USA; Cat. No. 420801).
- Intracellular Staining Permeabilization Wash Buffer (BioLegend, San Diego, CA, USA; Cat. No. 421002).
- Cell Staining Buffer (BioLegend, San Diego, CA, USA; Cat. No. 420201).
- 0.4% Trypan Blue solution (Abisense, Moscow, Russia; Cat. No. DYE-01-4-100ML).

### 2.6. Required Equipment

- Class II biological safety cabinet, model BMB-II-“Laminar-S”-1.2 NEOTERIC (Lamsystems, Miass, Russia; Cat. No. 1R-B.001-12).
- Microcentrifuge with a rotor for 1.5 mL tubes, model Mini Spin Plus (Eppendorf, Hamburg, Germany; Cat. No. 5453000015).
- CO_2_ incubator S-Bt Smart Biotherm or an alternative (Biosan, Riga, Latvia; Cat. No. BS-010425-A01).
- Automated cell counter, model LUNA-FL™ (Logos Biosystems, Inc., Anyang-si, Gyeonggi-do, Republic of Korea; Cat. No. L20001).
- Imaging flow cytometer, model Amnis FlowSight (CytekBiosciences, Fremont, CA, USA; Cat. No. 100220).

### 2.7. Required Software

- INSPIRE™ software v. 200.1.620.0 (CytekBiosciences, Fremont, CA, USA)—for data acquisition.
- IDEAS^®^ 6.2 software (CytekBiosciences, Fremont, CA, USA)—for imaging flow cytometry data analysis.
- FluoSta v.1.0—in-house-developed software for statistical analysis of IDEAS reports (Supplementary Materials, https://github.com/SirenOmica/FluoSta; date of accessed: 6 November 2025).
- Microsoft Excel 2019 (Microsoft, Redmond, WA, USA; optional)—for transfer and additional processing of statistical results.

### 2.8. Sample Preparation and Data Acquisition Using INSPIRE

**Figure 1:**
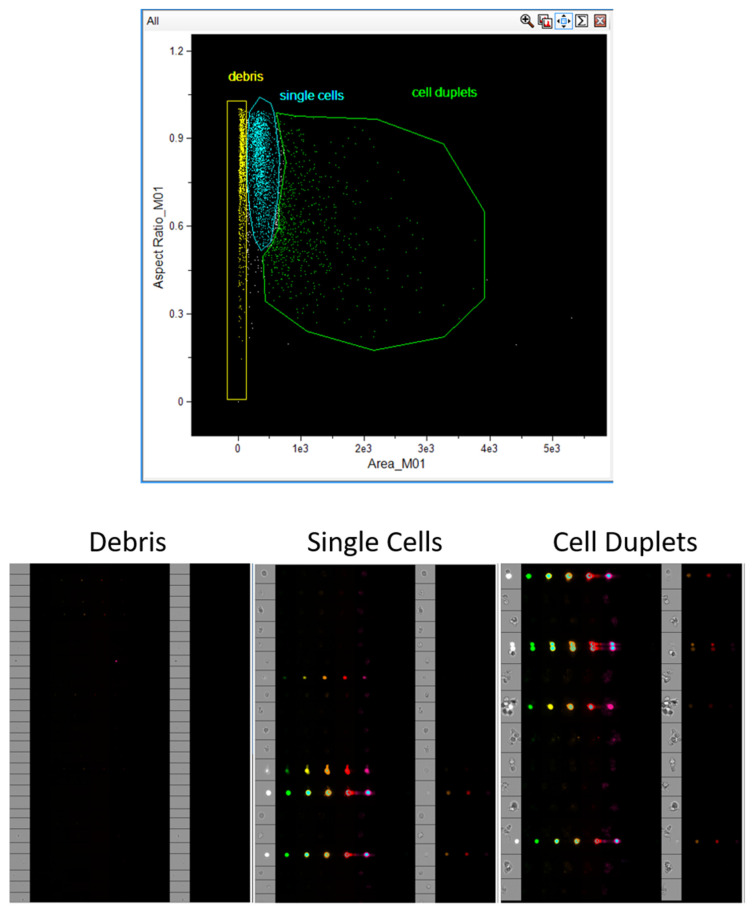
Event populations observed during data acquisition in INSPIRE (CytekBiosciences, Fremont, CA, USA). Debris, single cells, and cell doublets (clumps) are commonly observed. Subsequent analysis is based on the study of single cells; therefore, to analyze a greater number of these and increase the statistical power, it is recommended to acquire only this population in INSPIRE (CytekBiosciences, Fremont, CA, USA).

1. Incubate the test compound with cells in an appropriate culture medium. In this study, MT-4 cells were cultured in RPMI-1640 medium (Gibco, Waltham, MA, USA; Cat. No. 11875093) supplemented with 10% FBS (Gibco, Waltham, MA, USA; Cat. No. A5209502) and 0.001% PenStrep (Gibco, Waltham, MA, USA; Cat. No. 15140122) for 48 h. Aliquots (≈1 × 10^6^ cells) were collected after 1, 4, 12, 24, 36, and 48 h of incubation.
2. Power on the imaging flow cytometer and perform the initial startup and calibration procedures.
3. Transfer a cell sample into a 1.5 mL microcentrifuge tube (Eppendorf, Hamburg, Germany; Cat. No. 022364111). Centrifuge at 300 RCF for 5 min (centrifugation conditions may require optimization).
4. Carefully aspirate the supernatant. Resuspend the cell pellet in 500 µL of 1× DPBS (Gibco, Waltham, MA, USA; Cat. No. 14190144).
5. Centrifuge at 300 RCF for 5 min.
6. Aspirate the supernatant. Resuspend the cell pellet in 500 µL of 1× DPBS (Gibco, Waltham, MA, USA; Cat. No. 14190144).
7. Centrifuge at 300 RCF for 5 min.
8. Resuspend the final cell pellet in 100 µL of 1× DPBS containing 0.04% BSA (Thermo Fisher Scientific, Waltham, MA, USA; Cat. No. B14).
9. Optional: Add 5 µL of antibodies against surface antigens to the cell suspension. Incubate for 1 h at room temperature (optimized staining conditions, as per the antibody manufacturer’s protocol, are strongly recommended). Add 400 µL of 1× DPBS (Gibco, Waltham, MA, USA; Cat. No. 14190144) to the cells and resuspend. Centrifuge at 300 RCF for 5 min. Aspirate the supernatant. Resuspend the cells in 100 µL of 1× DPBS (Gibco, Waltham, MA, USA; Cat. No. 14190144) with 0.04% BSA (Thermo Fisher Scientific, Waltham, MA, USA; Cat. No. B14).
10. Optional: Add 400 mL of fixation buffer (BioLegend, San Diego, CA, USA; Cat. No. 420801) to the cells. Incubate the cells at room temperature for 10 min. Centrifuge at 300 RCF for 5 min. Aspirate the supernatant. Resuspend the cells in 500 µL of 1× Intracellular Staining Permeabilization Wash Buffer (BioLegend, San Diego, CA, USA; Cat. No. 421002). Incubate the cells at room temperature for 10 min. Centrifuge at 300 RCF for 5 min. Aspirate the supernatant. Resuspend the cells in 500 µL of Cell Staining Buffer (BioLegend, San Diego, CA, USA; Cat. No. 421002). Centrifuge at 300 RCF for 5 min. Aspirate the supernatant. Resuspend the cells in 100 µL of Cell Staining Buffer (BioLegend, San Diego, CA, USA; Cat. No. 421002). Add 1 µL of antibodies to clathrin heavy chain (Thermo Fisher Scientific, Waltham, MA, USA; Cat. No. MA1-065-A647) at a concentration of 10 µg/mL. Incubate the cells at room temperature in the dark for 1 h. Centrifuge at 300 RCF for 5 min. Aspirate the supernatant. Resuspend the cells in 100 µL of Cell Staining Buffer (BioLegend, San Diego, CA, USA; Cat. No. 421002).
11. Place all sample tubes in +4 °C. For data acquisition, remove tubes from the refrigerator one at a time to minimize the duration cells are exposed to room temperature.
12. For acquisition, take one tube. Add 2 µL of PI (Lumiprobe, Hunt Valley, MD, USA; Cat. No. 19010), mix gently, and incubate for 5 min while protected from light.
13. Place the tube containing the stained cell suspensiocn into the Amnis (CytekBiosciences, Fremont, CA, USA; Cat. No. 100220) sample loader. Acquire data for single, live cells (Figure 1). Live cells should exhibit no PI fluorescence signal (intensity level < 1 × 10^4^). We recommend collecting 3–5 technical replicates per cell suspension sample.

Note 1: It is critical to remember that subsequent analysis in IDEAS 6.2 requires a compensation matrix, for which compensation controls are essential. At the beginning or in the middle of the experiment, allocate an additional sample for the experimental fluorophore control (e.g., cells stained with the fluorescent chemical compound only) and an additional sample for the PI control (cells stained with PI only).

### 2.9. Analysis of Results in IDEAS 6.2

Note 1: An analysis template for IDEAS 6.2 (CytekBiosciences, Fremont, CA, USA) is provided in the Supplementary Materials. This template may require optimization for specific cell morphologies and other fluorescent labels used.

The provided template includes the following sequential gating steps:

1. Gating of in-focus cells. Cells that are not in focus cannot be accurately analyzed for fluorescence intensity.
2. Gating of the population passing through the interrogation point 20–30 s after the start of the acquisition. This step excludes potential carryover events from the previous sample.
3. Gating of single cells. Despite resuspension and a stable laminar flow, some events may represent two or more cells clumped together, which would be recorded as a single event with inaccurate fluorescence and morphology measurements.
4. Gating of live cells. Dead cells often exhibit high autofluorescence and the nonspecific uptake of chemical compounds.
5. Gating of the population of cells that have internalized the chemical compound versus the non-internalized population.
6. Creation of image masks to define the cytoplasmic region (AdaptiveErode (M01, Ch01, 70)) and the plasma membrane region (Object (M01, Ch01, Tight) and not AdaptiveErode (M01, Ch01, 70)).
7. Calculation of new parameters: Perimeter (M01, Ch01), Circularity (M01, Ch01), and Entropy (M02, Ch2, 1 µm).
8. Gating of cells exhibiting a fluorescent signal specifically within the cytoplasmic mask region.

### 2.10. Statisctical Analysis of Results in IDEAS 6.2

For the statistical analysis of the results, software (FluoSta v1.0) was developed using the R language v. 4.4.2 (R Foundation for Statistical Computing, 2024; Vienna, Austria). This software enables comprehensive processing of tabular results (in txt format generated by IDEAS) and performs a standard set of statistical tests and visualizations. The software outputs descriptive statistics of the data (mean with standard deviation (SD)) and comparative statistics. FluoSta indicates the significance of differences between all studied substances (one-way ANOVA with reporting of Fisher’s criterion (F-statistic))—in this case, between antisense oligonucleotides with different chemical modifications, as well as pairwise comparisons between specific chemical modifications (Tukey’s HSD test). The contribution of modifications to changes in the studied trait is investigated using omega-squared (ω^2^). The effect size for the difference between two modifications is estimated using Cohen’s d. For comparisons of samples within a single chronological time point, Repeated Measures ANOVA (RM-ANOVA) is used, followed by pairwise comparisons between time points using pairwise *t*-tests. The contribution of the incubation time to changes in the studied parameter is expressed using the generalized eta-squared (GES) measure. The effect size between two time points is assessed using Cohen’s dz. All pairwise comparisons are performed with the application of the Benjamini–Hochberg correction with a threshold value of *p* < 0.05.

### 2.11. Procedure for Using FluoSta v.1.0

1. Launch the installer (FluoStaInstaller.exe). Open the program and read the README file containing usage tips.
2. In the pop-up menu, select the statistical reports (.txt) generated by IDEAS 6.2 (CytekBiosciences, Fremont, CA, USA).
3. Specify the incubation time for each .txt file. Run the program. If necessary, change the order of time points in the “Order of time points” section.
4. The open window of FluoSta displays descriptive statistics, a comparative analysis, and interactive graphs for data visualization. You can use the statistical analysis results to compare parameters of a single compound over time (the RM-ANOVA and *t*-tests tab), as well as to compare different compounds at a single chronological time point (the ANOVA and Tukey tab). In the output, you can download a file in .xlsx format with your statistical analysis. Two display modes are available for the graphs. If needed, graphs can be downloaded in PNG format by clicking the camera icon.

Note 1: Statistical tests for multiple comparisons may yield incorrect results with low data variance; therefore, we recommend using at least three biological replicates to avoid this situation.

Note 2: The statistical analysis will be incomplete if for any compound, there are not at least two biological replicates present at each chronological time point (it is preferable to avoid the complete absence of a compound at any time point).

Note 3: Flow cytometry output data files (.daf) must have the same name throughout the experiment so that the software can identify them as a single sample for statistical analysis. For example, files for the first biological replicate of cells incubated with the 3′LNA antisense oligonucleotide should be named 3′LNA_1 throughout the experiment, while the second biological replicate should be named 3′LNA_2, etc. Do not include a space or the incubation time in the .daf file name; instead, indicate the chronological point in the final TXT report. You will be able to assign the incubation time within the program via a pop-up window after loading the statistical reports (.txt).

## 3. Results

### 3.1. Data Acquisition in INSPIRE

The generation of raw image files (.rif) from cell imaging on the Amnis FlowSight (CytekBiosciences, Fremont, CA, USA) is not the most labor-intensive stage; however, it can significantly streamline subsequent analysis and enhance the statistical power of the study through effective gating of cells relevant for downstream processing. This requires the acquisition of single cells, excluding debris and doublets. If compound toxicity is being assessed in your experiment by an independent method, such as an MTT assay, it is preferable to acquire single, live cells. The gating strategy for identifying single cells does not require a compensation matrix and is based on a plot of the cell area versus the aspect ratio (Figure 1).

**Figure 2:**
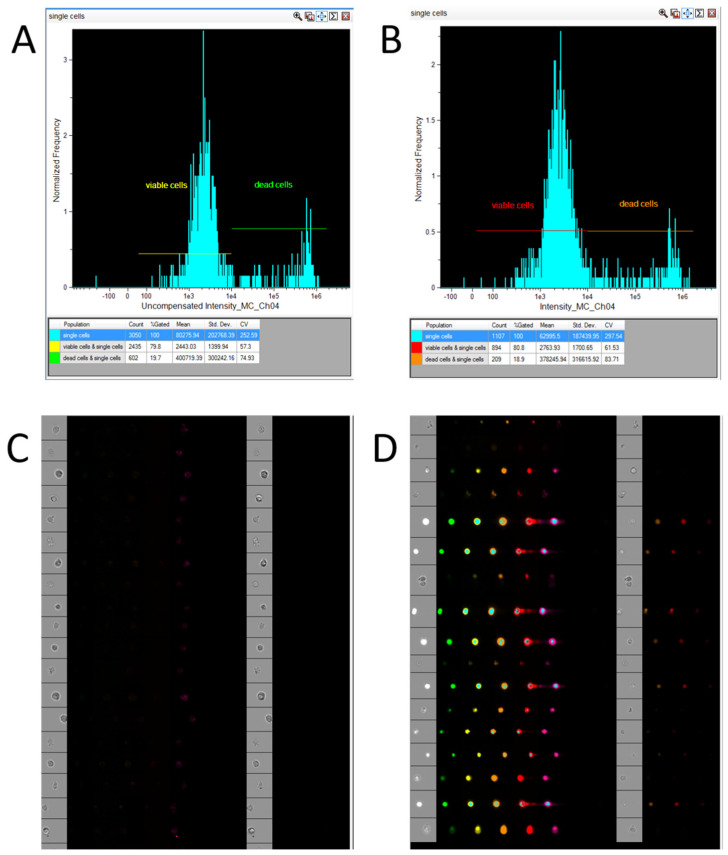
Acquisition of single, live cells in INSPIRE (CytekBiosciences, Fremont, CA, USA): (**A**)—gating of the live cell population (PI-negative) without a compensation matrix; (**B**)—gating of the live cell population (PI-negative) using a compensation matrix; (**C**)—image gallery of the single, live cell population acquired in INSPIRE (CytekBiosciences, Fremont, CA, USA); (**D**)—image gallery of the dead cell population acquired in INSPIRE (CytekBiosciences, Fremont, CA, USA).

It is preferable to acquire not just single cells, but single, live cells. An exception to this is situations where it is necessary to assess the compound toxicity using imaging flow cytometry. Single, live cells can be gated with or without a PI compensation matrix (Figure 2).

In our experiment, visualizing the plots during acquisition of live cells with and without a compensation matrix had a negligible impact on the proportion of gated viable cells: 78.9% of all single cells without compensation versus 80.8% when using the compensation matrix (see Figure 2). The compensation matrix for this training dataset can be found in the attached files. As described in the Section 2, in this study, two fluorescent dyes were used: FAM (excitation/emission ~494/518 nm) and propidium iodide (PI) (~535/617 nm). To correct for spectral overlap between the FAM and PI detection channels, a compensation matrix was applied, as calculated using the INSPIRE software v. 200.1.620.0 based on single-stain controls stained with either FAM or PI only. Since the extinction/emission and optimal concentrations of fluorophores can vary significantly, researchers applying this pipeline with other dyes or under different experimental conditions will need to generate their own specific compensation matrix using appropriate single-stain controls. At the same time, the developed pipeline does not interfere with the use of multiple dyes and multiplexing. However, the user will have to optimize the next stage (the IDEAS template) to output populations of cells stained with dyes that are used by the user. For example, instead of FAM dye, the user will use HEX dye; instead of PI dye, DAPI dye.

### 3.2. Analysis of Results in IDEAS

Implementation of the developed imaging flow cytometry data analysis pipeline enabled robust differentiation between the localization of fluorescently labeled antisense oligonucleotides associated with the plasma membrane and signal residing within the cytoplasm. The provided analysis template for IDEAS (Supplementary Materials) can be adapted for specific cell types and fluorophores. We also provide tutorial materials, including several datasets featuring compound internalization studies and investigations of HIV cytopathic effects. While the template is specifically optimized for internalization research, the open-source statistical processing code is compatible with any experimental design.

**Figure 3:**
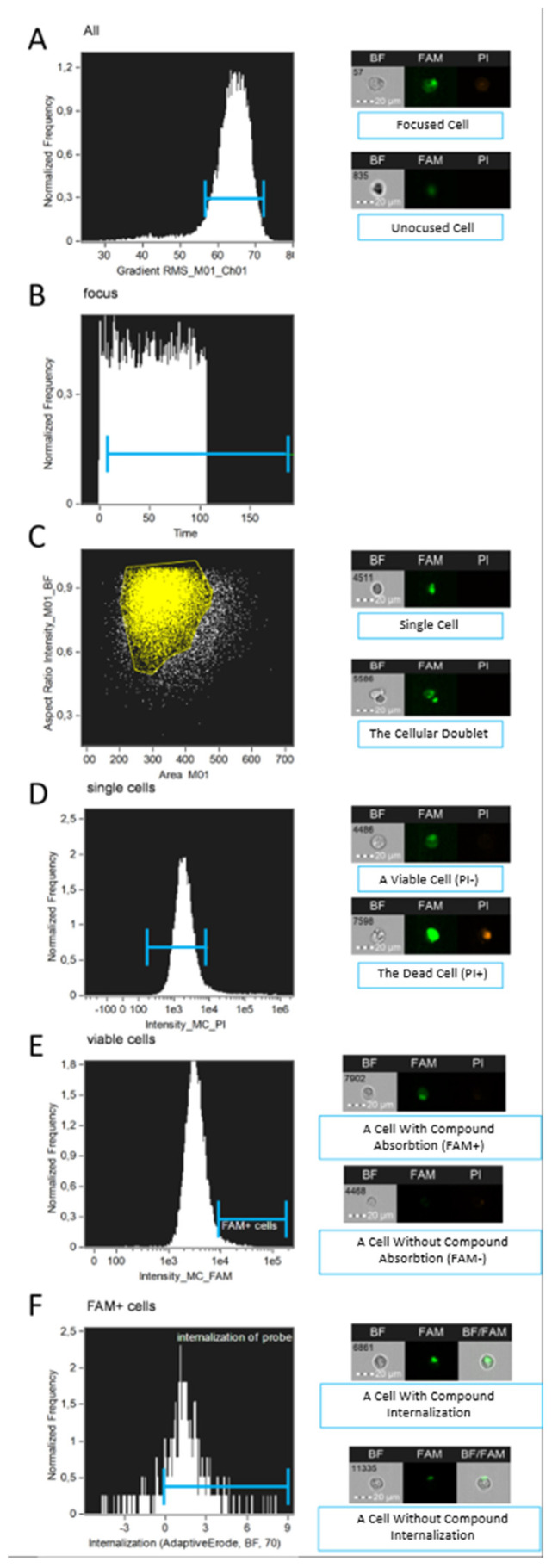
Gating strategy for cell populations applied to analyze chemical compound internalization using the proposed IDEAS (CytekBiosciences, Fremont, CA, USA) template: (**A**)—selection of in-focus cells; (**B**)—selection of cells passing through the flow cell after 20–30 s from the start of the .rif file acquisition; (**C**)—selection of single cells; (**D**)—selection of viable cells; (**E**)—selection of cells with compound absorption (membrane-associated or cytoplasmic); (**F**)—selection of cells with compound internalization, that is selecting cells on the graph Max Pixel versus Intensity, where cells with a coefficient > 0 are considered cells having a fluorescent signal within the cytoplasmic mask. The internalization coefficient can be defined as the logarithmic ratio of the intensity inside the cell to the intensity of the entire cell.

Analysis of raw image files (.rif) quantifies the following: the number of viable single cells that have absorbed the compound (either membrane-associated or cytoplasmic), the proportion of cells with internalized probe, and the entropy of the compound’s distribution within the cytoplasm. The gating strategy is illustrated in Figure 3.

**Figure 4:**
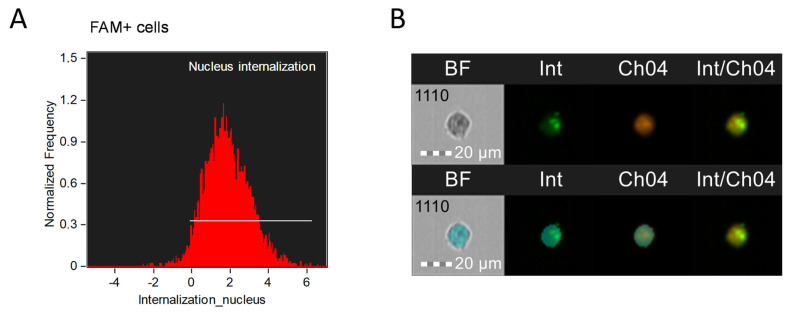
Investigation of the internalization of a chemical compound into the nucleus (karyoplasm) of MT-4 cells using the Internalization parameter via Int (green) and PI (orange) fluorescence channels: (**A**)—graph of evaluation of internalization of antisense oligonucleotides into the nucleus; (**B**)—images of cells with internalized FAM signal in the karyoplasm. The created Nucleus mask (Erode, M04, 3) is superimposed on the image below. The cells were stained with antisense oligonucleotides with a fluorescent label FAM (Int-FAM) and propidium iodide (PI) to assess the cell viability. Fluorescence was excited using a 488 nm laser with a power of 60 mW. The green fluorescence from FAM was detected in the second channel using a 532/55 nm filter, while the orange fluorescence from PI was detected in the third channel using a 577/35 nm filter. The images were captured at a total magnification of 20× (lens numerical aperture = 0.6), with a pixel size of 1 × 1 µm.

Some compounds exert their effects in specific cellular compartments, such as the nucleus. IDEAS 6.2 allows you to determine the location of the label in individual parts of the cell. For the nucleus, as for a large structure, the Internalization parameter with the creation of a “Nucleus” mask (Erode, M04, 3) is more suitable by analogy with a cytoplasmic mask (Figure 4).

**Figure 5:**
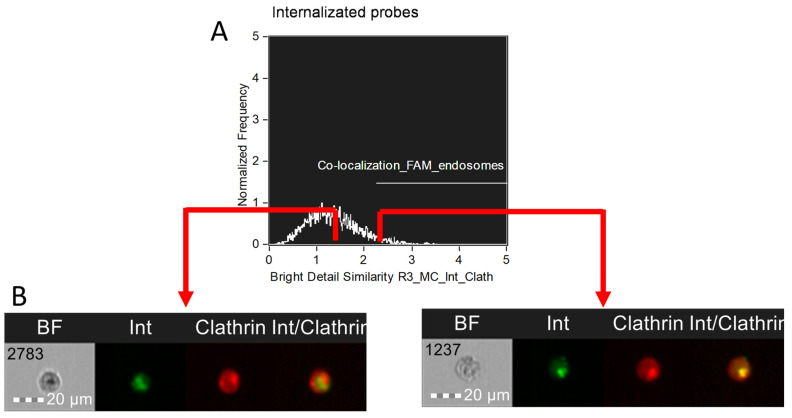
Investigation of the co-localization of a chemical compound into the endosomes of MT-4 cells using the Internalization parameter via Int-FAM (green) and Clathrin-AF647 (red) fluorescence channels: (**A**) graph of co-localization of fluorescent labels associated with antisense oligonucleotide Int (FAM) and clathrin, which is involved in the stabilization of endosomes (AF647); (**B**) images of cells without colocalization of Int-FAM and Clathrin-AF647 signals and with colocalization of Int-Fam and Clathrim-AF647 signals. The cells were stained with antisense oligonucleotides with a fluorescent label FAM (Int-FAM) and antibodies against clathrin were conjugated with Alexa Fluor 647 (AF647). Fluorescence was excited using a 488 nm laser with a power of 60 mW and a 642 nm laser with a power of 60 mW. The green fluorescence from FAM was detected in the second channel using a 532/55 nm filter, while the red fluorescence from AF647 was detected in the eleventh channel using a 702/85 nm filter. The images were captured at a total magnification of 20× (lens numerical aperture = 0.6), with a pixel size of 1 × 1 µm.

Smaller structures, such as endosomes, will require a different instrument since they are too small and the resolution of the Amnis cameras will not be sufficient to visualize individual endosomes and their membranes. To evaluate localization in small compartments, it is better to use the basic Wizard “Co-localization”, available in IDEAS 6.2 (Figure 5). This Wizard uses the Bright Detail Similarity R3 parameter, which is the log-transformed Pearson’s correlation coefficient of the localized bright spots with a radius of 3 pixels or less within the masked area in the two input images. Since the bright spots in the two images are either correlated (in the same spatial location) or uncorrelated (in different spatial locations), the correlation coefficient varies between 0 (uncorrelated) and 1 (perfect correlation) and does not assume negative values.

**Figure 6:**
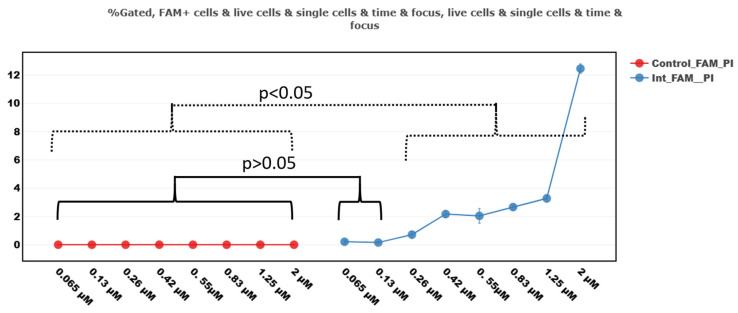
The number of FAM-positive cells incubated with different concentrations (0.065–2 µM) of Int ASO conjugated with FAM fluorescent dye. The comparison was carried out with control samples that contained a nutrient medium with cells without the addition of antisense oligonucleotide. The lowest concentration of the antisense oligonucleotide Int-FAM, at which FAM-positive cells are still detected, is 0.26 µM. The graph was compiled using the FluoSta v.1.0 software.

We additionally evaluated the sensitivity of the proposed method for assessing the absorption of antisense oligonucleotides conjugated with FAM. According to the results of this assessment, the sensitivity limit is in the range of ≈0.26 µM of the substance (Figure 6). However, it should be borne in mind that the sensitivity depends on the fluorescent labels used, the parameters of the compound itself, and the cell culture.

**Figure 7:**
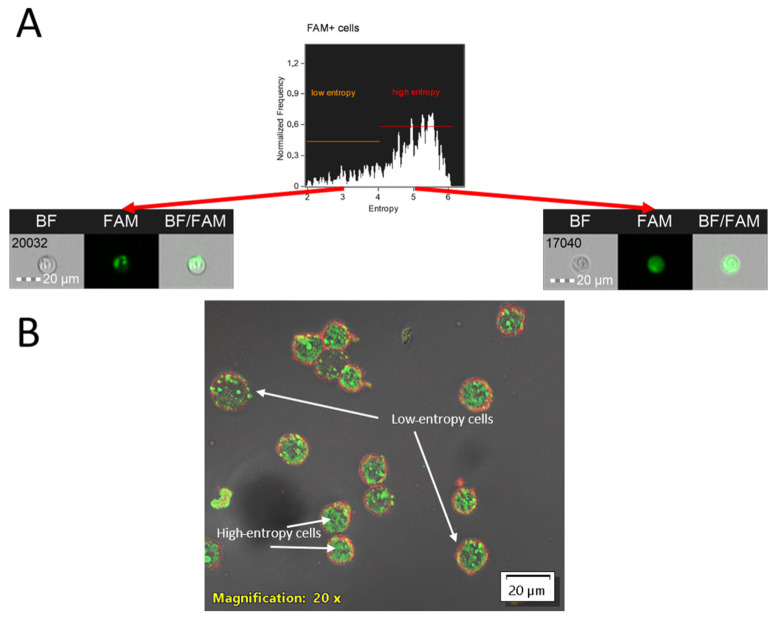
Analysis of internalized compound entropy in an MT-4 cell culture following a 4 h incubation with PS-modified antisense oligonucleotide Int conjugated to FAM: (**A**) entropy plot generated using the Entropy parameter within the cytoplasmic mask, accompanied by representative imaging flow cytometry images of cells exhibiting low and high entropies. Images were acquired at 20× magnification (NA = 0.6) on an Amnis FlowSight imaging flow cytometer (CytekBiosciences, Fremont, CA, USA); (**B**) confocal microscopy image of MT-4 cell culture following a 4 h incubation with a PS-modified antisense oligonucleotide (Int) conjugated to FAM. The image was acquired using an Olympus FV3000 confocal microscope (Olympus, Tokyo, Japan) with a UPLSAPO20X (Olympus, Tokyo, Japan)objective at 20× magnification (NA = 0.75). The image acquisition utilized 488 nm (FAM, 10% power) and 642 nm (AF700, BF, 50% power) lasers. Detection was performed using high-sensitivity detectors (HSD1 and HSD2) at 500 V. Scale bar is located in the lower-right corner of images (20 µm size). Cells with low and high ASO-FAM fluorescence entropies are indicated by arrows with corresponding labels.

Based on the final parameter, an internalization coefficient is calculated, defined as the logarithmic ratio of fluorescence intensity in the cytoplasmic mask region to the fluorescence of the entire cell (base “Object” mask) [9]. To assess the distribution of the chemical compound within the cytoplasm, the Shannon entropy parameter is used: higher values indicate a more uniform signal distribution without areas where the fluorescence intensity is markedly stronger than the average (see Figure 7).

The entropy parameter, as one of Haralik’s texture statistics, is calculated based on the Gray Level Co-occurrence Matrix (GLCM). To calculate it, the source images are first converted to 8-bit to reduce the noise, then the intensity distribution in neighboring pixels at a given distance (granularity) is analyzed for each pixel, and based on this distribution, a measure of signal heterogeneity—entropy—is calculated. The mathematical definition of entropy is as follows:

$$\mathrm{Entropy}=\;-\sum_{i-0}^{N-1}\sum_{j-0}^{N-1}p_{\mathrm{ij}}\log_{2}(p_{\mathrm{ij}})$$

where I and j—brightness level indexes (from 0 to N − 1); p(i,j)—a segment of the normalized matrix of spatial dependencies of brightness levels (GLCM), representing the probability of the joint appearance of brightness levels i and j for two pixels separated by a given distance and direction; and N—number of distinct gray levels in the quantized image [10].

**Figure 8:**
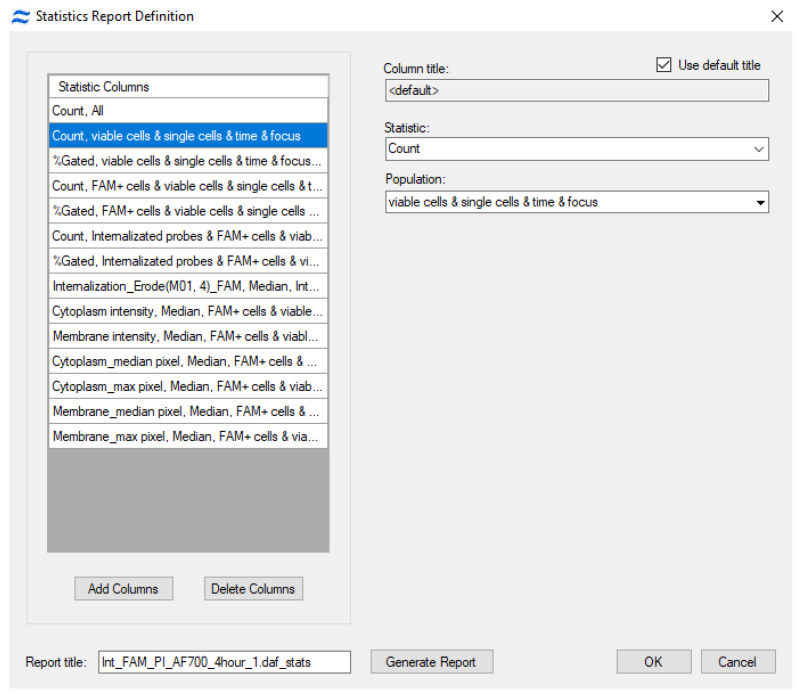
The statistical report generation menu in IDEAS 6.2 (CytekBiosciences, Fremont, CA, USA). In this menu, it is necessary to select parameters—such as the count and proportion of cells in various populations, morphological characteristics, and fluorescence intensity in specific populations—that will be meaningful for formulating biologically relevant conclusions.

The final step in IDEAS involves generating a statistical report. To accomplish this, navigate to the menu tabs Report > Define statistic report and select the parameters specified in the IDEAS template. These parameters include total cell count, count of viable single cells (Count, viable cells & single cells & time & focus) and their percentage, count of cells with signal absorption, percentage of cells with signal absorption, and median entropy (Figure 8).

Once the statistical report is compiled, IDEAS generates a .txt file, which should be named according to the chemical compound and incubation period analyzed. Each .txt file must contain parameter data for all compounds studied at a single timepoint. As part of the developed pipeline, these .txt files are subsequently imported into the statistical analysis software.

**Figure 9:**
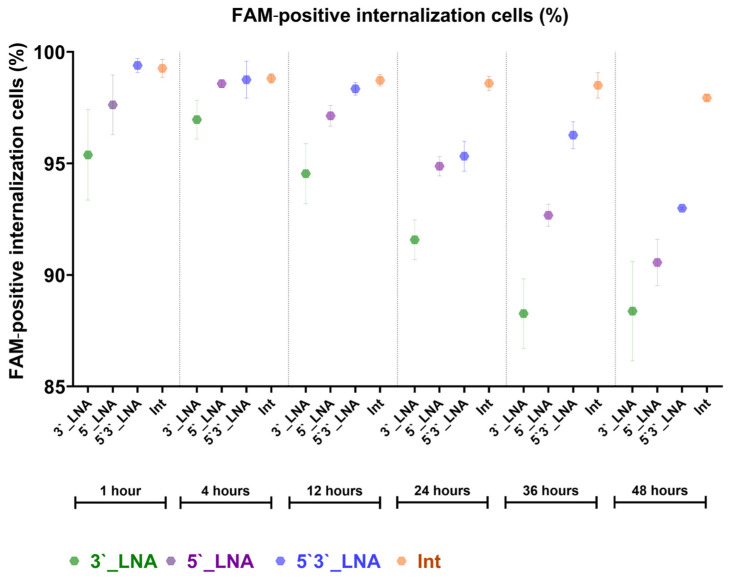
The results of the assessment of cellular internalization of ASO with antiretroviral activity following different incubation times (1, 4, 12, 24, 36 and 48 h) obtained using the developed template for IDEAS (CytekBiosciences, Fremont, CA, USA). The graph is built in FluoSta v.1.0 software.

In our internalization time-course study with PS- and PS/LNA-modified antisense oligonucleotides in the MT-4 cell line, the developed pipeline enabled detection of significant differences between the compounds. The PS-modified antisense oligonucleotides demonstrated faster and more efficient internalization compared with the PS/LNA-modified nucleotides (Figure 9).

These antisense oligonucleotides were not complexed with any delivery vehicles, so their bioavailability was determined exclusively by their chemical properties. It is recognized that LNA modification enhances cytoplasmic stability via improved nuclease resistance, though its stereochemical properties may also affect the membrane permeability.

Additionally, high entropy values for the ASO-FAM fluorescent signal in our study revealed the ability of the ASO to spread throughout the cytoplasmic content, reflecting endocytic entry and subsequent partial degradation. Upon degradation, antisense oligonucleotides retain their fluorescent signal within endosomes, resulting in regions of locally elevated fluorescence intensity, and thus, low entropy. However, if the compound’s target is a specific cellular compartment or organelle, high entropy values indicate impaired delivery to the intended destination.

### 3.3. Validation of Imaging Flow Cytometry Results by Confocal Microscopy

To verify the results obtained with imaging flow cytometry, laser scanning confocal microscopy was employed using ASO-FAM and CD4-AF700 as fluorescent markers. Quantitative comparisons—including the number of cells absorbing the compound and the number exhibiting probe internalization—revealed no statistically significant differences (*p* > 0.05) between the two methods. For example, following a 4 h incubation with the antisense oligonucleotide Int, the percentage of cells displaying probe internalization was 97.4% (SD ± 0.131), as measured by imaging flow cytometry, compared with the 93.6% (SD ± 0.74) obtained by manual cell counting in confocal micrographs (*p* < 0.05).

**Figure 10:**
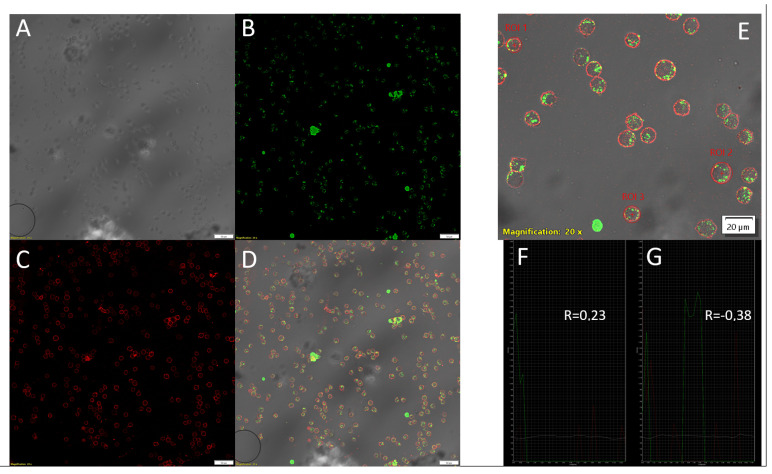
Confocal microscopy of an MT-4 cell culture following a 4 h incubation with PS-modified antisense oligonucleotide Int conjugated to FAM. Images were acquired using an Olympus FV3000 confocal microscope (Olympus, Tokyo, Japan) with a UPLSAPO20X (Olympus, Tokyo, Japan) objective at 20× magnification (NA = 0.75). The image acquisition utilized 488 nm (ASO-FAM, 10% power) and 642 nm (CD4-AF700, BF, 50% power) lasers. Detection was performed using high-sensitivity detectors (HSD1 and HSD2) at 500 V. Scale bars are located in the lower-right corners of images: 50 µm (images (**A**–**D**)) and 20 µm (image (**E**)). (**A**) Pseudo-brightfield channel; (**B**) ASO-FAM fluorescence channel; (**C**) CD4-AF700 fluorescence channel; (**D**) composite image of BF, ASO-FAM, and CD4-AF700 channels; (**E**) image fragment with regions of interest (ROIs), including a cell without probe internalization (ROI1), a cell with probe internalization (ROI2), and a cell with partial internalization of absorbed ASO (ROI3); (**F**) one-dimensional fluorescence profile of the cell in ROI1 showing the Pearson correlation R coefficients for ASO-FAM and CD4-AF700 colocalization; (**G**) one-dimensional fluorescence profile of the cell in ROI2 showing Pearson correlation R coefficients for ASO-FAM and CD4-AF700 colocalization.

Confocal microscopy was also used to confirm probe internalization in cells that visually exhibited intracellular signal localization. For this purpose, we measured the 1D fluorescence intensity profiles of cells with visually confirmed internalized signals and those without (Figure 10).

It should be noted that the fluorescent signal in cells without internalization does not extend beyond the “framework” of the fluorescent signal from AF700 conjugated to CD4 (Figure 10F,G). Furthermore, we quantified the colocalization of ASO-FAM and CD4-AF700 signals using Pearson’s correlation coefficient (R): in cells without compound internalization, this coefficient was positive, indicating that ASO molecules were located adjacent to CD4, i.e., near the cytoplasmic membrane. Conversely, cells exhibiting pronounced compound internalization showed negative R values, indicating fewer ASO-FAM fluorescent signals were adjacent to the CD4-AF700 signals (Figure 10F,G).

### 3.4. Statistical Analysis in FluoSta v.1.0

The final stage of the pipeline involves utilizing the developed FluoSta software (v.1.0), which facilitates the generation of descriptive statistics, comparisons between different compounds at individual time points, and longitudinal comparisons of parameters for a single compound across multiple time points.

FluoSta is implemented in R (utilizing the following packages: shiny, shinybusy, openxlsx, ez, dplyr, effectsize, plotly, RColorBrewer) and is available in two formats: as an executable installer (incorporating the appropriate R version and all necessary libraries) or as an R script executable within RStudio v. 2025.09.2+418.

**Figure 11:**
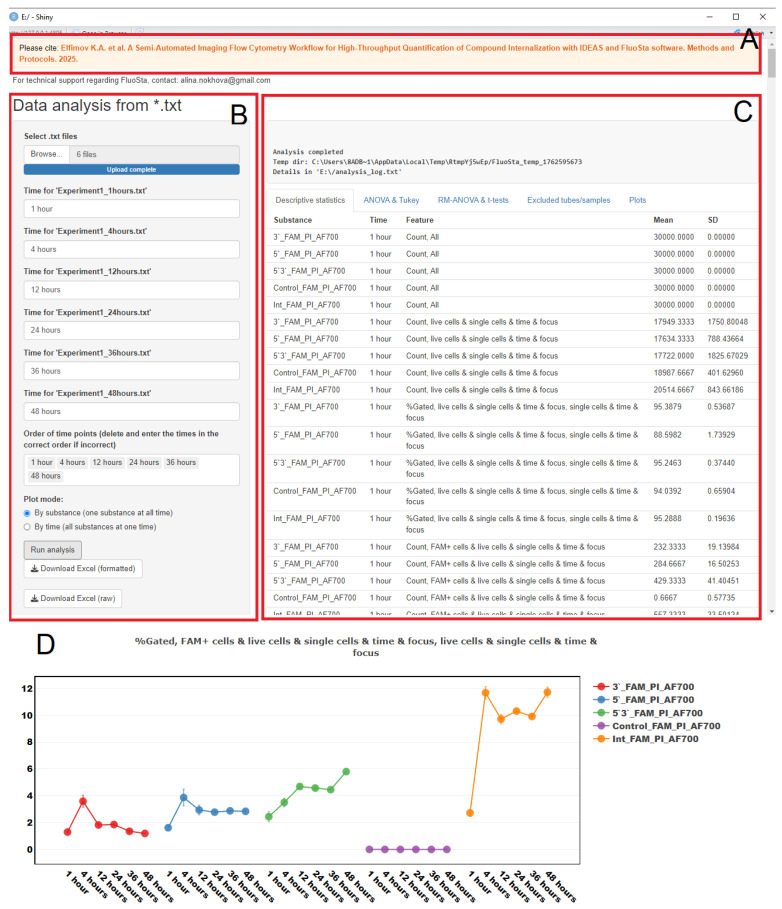
Interface of the developed FluoSta v.1.0 software for statistical analysis of imaging flow cytometry results: (**A**) citation information; (**B**) file upload menu (Browse button) with incubation time assignment for each statistical report (Time for <…>) and plot type selection (for each compound over time or for all compounds at individual time points). The “*” symbol displays the name of the user’s file.; (**C**) menu containing descriptive statistics (Descriptive statistics), comparison results for all samples at individual time points (ANOVA & Tukey), comparison results for a single sample over time (RM-ANOVA & *t*-tests), excluded records (e.g., due to lack of variance or lack of data; Excluded tubes/samples), and scatter plots with standard deviations (Plots); (**D**) example of a graph constructed by FluoSta for MT-4 cells with AOS-FAM signal absorption.

Prior to launching the program, we recommend reviewing the README.txt file containing the usage guidelines. This document includes information about training datasets, interpretation of calculated metrics, and the necessity of maintaining consistent naming conventions for compounds and their biological replicates throughout the experiment to enable FluoSta to identify them as a unified sample (refer to Materials and Methods, Statistical Analysis in Rstudio, Note 3). The program’s graphical interface (opens in a web browser) comprises several sections (Figure 11).

The application loads tabular *.txt files (one file per time point) containing a “File” column. This column contains entries with the expected object name in the format <object>_<Num>.daf, where <object> represents the object name (which must remain identical for each object throughout the experiment across all time points and text files, without a space in the name), and <Num> indicates the tube number. The software subsequently calculates statistical metrics and generates plots.

Descriptive statistics (under “Descriptive statistics” tab) include means and SD for each object × time combination. The “ANOVA & Tukey” tab performs comparisons between groups/modifications/objects at each investigated time point. This analysis calculates Fisher’s criterion from one-way ANOVA with effect significance estimation using ω^2^ (proportion of trait variance explained by the “modification” factor) with a 95% confidence interval (CI). For pairwise comparisons between groups, statistical significance is assessed using Tukey’s HSD test, while the effect size between groups is reported as Cohen’s d.

For comparisons of different time points within a single group, RM-ANOVA is employed with GES calculation (proportion of trait variation explained by the “incubation time” factor) with a 95% CI. Comparisons between two time points utilize pairwise *t*-tests and Cohen’s dz as effect size measure.

The “Excluded tubes/samples” tab contains a list of tubes excluded from comparative analysis (with reasons for exclusion: absence of tubes at certain time points, zero variance, insufficient data, etc.). Analysis results are exported to Excel (Results.xlsx and Results_raw.xlsx).

The statistical analysis performed by FluoSta v.1.0 includes descriptive statistics reporting mean values with their SD, ANOVA with Tukey’s HSD test for identifying differences between different compounds (samples), and RM-ANOVA with a pairwise *t*-test for detecting differences in parameters of a single sample over time. Additionally, the program generates dot plots based on provided results, displaying means with SD and incubation time. FluoSta v.1.0 operates without requiring an internet connection.

## 4. Discussion

The proposed analytical pipeline for investigating chemical compound internalization, based on imaging flow cytometry, integrates the complementary strengths of conventional flow cytometry (high statistical power through analysis of large cell populations) and microscopy (precise signal localization information). This methodological approach is consistent with several previously reported strategies. For instance, Smirnov et al. developed a protocol quantifying Neisseria gonorrhoeae uptake by neutrophils using imaging flow cytometry, where simultaneous intracellular and bacterial labeling enabled automated discrimination between cells without bacteria, cells with non-colocalized signals, and cells with colocalized signals indicating bacterial internalization [7]. Similarly, Ofir-Birin et al. employed imaging flow cytometry to monitor uptake of malaria parasite-derived exosomes by target cells, quantifying fluorescently labeled extracellular vesicles as intracellular “spots” and calculating the percentage of cells with internalization using comparable gating strategies and biologically relevant features [6].

Our workflow expands upon these principles through the creation of cytoplasmic and membrane masks and calculation of the fluorescence ratio within the cytoplasmic mask relative to the total cellular fluorescence. Furthermore, we introduce a novel signal distribution entropy parameter to quantitatively assess the uniformity of chemical compound distribution within the cytoplasm. Unlike conventional flow cytometry, imaging flow cytometry enables clear differentiation between surface-adsorbed and internalized signals. This capability has been demonstrated in nanoparticle uptake studies where the computational separation of extracellular and intracellular regions allowed accurate calculation of internalization coefficients without requiring chemical surface washing [11], a principle similarly implemented in our approach.

Confocal microscopy provides superior spatial resolution for precise subcellular localization, making it ideal for detailed visualization of receptor internalization (e.g., G protein-coupled receptors) and subsequent intracellular trafficking in individual cells [12]. Imaging flow cytometry effectively bridges these methodologies by combining the statistical robustness of conventional flow cytometry with the spatial localization capabilities of microscopy.

A key feature of our pipeline is the developed FluoSta v1.0 application, which automates the statistical analysis of imaging flow cytometry data. The software imports IDEAS-generated reports, computes descriptive statistics, performs ANOVA with pairwise post hoc comparisons, and generates publication-quality visualizations. All processing occurs locally on standard personal computers without internet dependency, ensuring accessibility, reproducibility, and data security for users without programming expertise. FluoSta v1.0 significantly simplifies identification of statistically significant differences between experimental conditions while accounting for temporal dynamics and biological replication, thereby enhancing the interpretative power and scope of our analytical framework.

Notwithstanding these advantages, several limitations warrant consideration. The relatively limited availability of imaging flow cytometers in routine laboratory settings may restrict immediate widespread adoption. Although developed for the Amnis platform, some parts of our pipeline are not available for other flow cytometry or fluorescence microscopy platforms (template for IDEAS). Researchers would need to recreate equivalent masking and gating strategies using the native software of their respective platforms (e.g., IDEAS for Amnis). The specific algorithms for creating masks (e.g., AdaptiveErode, Object) may have different names or implementations, requiring optimization to achieve a comparable segmentation of cytoplasmic and membrane regions. However, the FluoSta statistical data processing program does not require the use of statistical reports generated by IDEAS for its work. For this reason, it can be used to analyze data obtained on any platform and by any method. Additionally, the IDEAS analysis template may require optimization for specific cell morphologies, particularly regarding cytoplasmic mask generation. The entropy parameter additionally requires careful consideration of fluorescence spot dimensionality, which varies substantially between large molecular complexes (spanning multiple pixels) and small chemical compounds such as antisense oligonucleotides (exhibiting minimal fluorescence from individual molecules).

## 5. Conclusions

We developed an analytical pipeline for quantifying compound internalization using imaging flow cytometry. This approach is distinguished by its comprehensive and semi-automated analysis, uniquely combining the high statistical power of conventional flow cytometry (through analysis of thousands of cells) with high-content spatial information on fluorescent label localization. The proposed IDEAS analysis template, together with our dedicated FluoSta v.1.0 software for statistical processing, enables rapid and reliable data interpretation. This integrated solution ensures accessibility for early-career researchers who may not be fully familiar with the advanced functionalities of IDEAS or specialized statistical methods.

## Figures and Tables

**Table 1:** Oligonucleotides investigated for their cellular internalization capacity. S, phosphorothioate linkage; L, locked nucleic acid (LNA) modification; [FAM], 6-carboxyfluorescein fluorophore label.

Oligonucleotide Designation	5′–3′ Sequence
FAM-Int III	[FAM]-C^S^T^S^T^S^G^S^A^S^C^S^T^S^T^S^T^S^G^S^G^S^G^S^G^S^A^S^T^S^T^S^G^S^T^S^A^S^G^S^G^S^G
FAM-Int III_3′-LNA	[FAM]-C^S^T^S^T^S^G^S^A^S^C^S^T^S^T^S^T^S^G^S^G^S^G^S^G^S^A^S^T^S^T^S^G^L^T^L^A^L^G^L^G^L^G
FAM-Int III_5′-LNA	[FAM]-^L^C^L^T^L^T^L^G^L^AC^S^T^S^T^S^T^S^G^S^G^S^G^S^G^S^A^S^T^S^T^S^G^S^T^S^A^S^G^S^G^S^G
FAM-Int III_5′/3′-LNA	[FAM]-^L^C^L^T^L^T^L^G^L^AC^S^T^S^T^S^T^S^G^S^G^S^G^S^G^S^A^S^T^S^T^S^G^L^T^L^A^L^G^L^G^L^G

## Data Availability

Data is contained within the article or Supplementary Materials.

## Supplementary Materials

The following supporting information can be downloaded from https://www.mdpi.com/article/10.3390/mps8060138/s1 and https://doi.org/10.5281/zenodo.17231142 (accessed on 6 November 2025): compensation matrix file for Example dataset 1—compensation_matrix_dataset1.ctm; compensation matrix file for Example dataset 2—compensation_matrix_dataset2; an IDEAS template for studying the internalization of chemical compounds—Main_template.ast; an IDEAS template Example dataset 2—template_dataset2.ast; raw (.rif) Amnis files from Example dataset1 and Example dataset 2; FluoSta .exe installer.
